# Effect of Cerium (IV) Oxide Particle Size on Polydimethylsiloxane Polymer to Form Flexible Materials against Ionizing Radiation

**DOI:** 10.3390/polym15132883

**Published:** 2023-06-29

**Authors:** Haifa M. Almutairi, Wafa M. Al-Saleh, Mohammad Ibrahim Abualsayed, Mohamed Elsafi

**Affiliations:** 1 Medical Physics Department, Umm Al-Qura University, Prince Sultan Bin Abdul-Aziz Road, Mecca P.O. Box 715, Saudi Arabia; hmmutayri@uqu.edu.sa; 2 College of Science and Health Professions, King Saud bin Abdulaziz University for Health Sciences, Al-Ahsa P.O. Box 6664, Saudi Arabia; salehw@ksau-hs.edu.sa; 3 King Abdullah International Medical Research Center, Al-Ahsa P.O. Box 3660, Saudi Arabia; 4 Department of Physics, Faculty of Science, Isra University, Amman 11622, Jordan; 5 Physics Department, Faculty of Science, Universiti Teknologi Malaysia, Skudai 81310, Malaysia; 6 Physics Department, Faculty of Science, Alexandria University, Alexandria 21511, Egypt

**Keywords:** polydimethylsiloxane, CeO_2_-nanoparticles, radiation shielding, attenuation coefficient

## Abstract

This study aims to investigate the impact of CeO_2_ content and particle size on the radiation shielding abilities of polydimethylsiloxane, also known as silicon rubber (SR). We prepared different SR samples with 10, 30, and 50% of micro and nano CeO_2_ and we measured the linear attenuation coefficient (LAC) for these samples. We found that the LAC of the SR increases by increasing the CeO_2_ and all prepared SR samples had higher LACs than the pure SR. We examined the effect of the size of the particles on the LAC and the results demonstrated that the LAC for nano CeO_2_ is higher than that of micro CeO_2_. We investigated the half value layer (HVL) for the prepared SR samples and the results revealed that the SR with 10% micro CeO_2_ had a greater HVL than the SR with 10% nano CeO_2_. The HVL results demonstrated that the SR containing nanoparticles had higher attenuation effectiveness than the SR with micro CeO_2_. We also prepared SR samples containing CeO_2_ in both sizes (i.e., micro and nano) and we found that the HVL of the SR containing both sizes was lower than the HVL of the SR with nano CeO_2_. The radiation protection efficiency (RPE) at 0.059 MeV for the SR with 10% micro and nano CeO_2_ was 94.2 and 95.6%, respectively, while the RPE of SR containing both sizes (5% micro CeO_2_ + 5% micro CeO_2_) was 96.1% at the same energy. The RPE results also indicated that the attenuation ability was improved when utilizing the micro and nano CeO_2_ as opposed to the micro CeO_2_ or nano CeO_2_ at 0.662, 1.173, and 1.333 MeV.

## 1. Introduction

Ionizing radiation can leave permanent damage on the human body as a result of the high energy of the photons that it consists of. Radiation, on the other hand, is of tremendous use in a variety of fields, including medicine, scientific research, industry, and agriculture. To ensure that the potential risks of radiation are not outweighed by its beneficial applications, appropriate precautions must be taken to reduce the negative effects of radiation as much as possible. In order to achieve this goal, the concepts of time (which include reducing the total amount of time spent in contact with radiation), distance (which involves maximizing the distance from the origin of the radiation), and the application of radiation shields are utilized [1,2,3]. A radiation shield is any substance that is employed to attenuate radiation and acts as a barrier between the source of the radiation and a person who is being protected by the shield. When selecting a shield to serve a particular function, numerous variables are taken into consideration in order to decide on the material that will be ideal for performing that function. For example, concrete has been shown to be quite efficient against X-rays and neutrons, making it an excellent material to employ as a liner for the walls of rooms containing machinery that utilizes radiation. In certain situations, concrete can be an excellent barrier; however, the material’s drawbacks, such as its tendency to crack and that it is unable to be moved, make it ineffective for a variety of other applications [4,5]. The most common radiation shielding material is lead, as well as products containing lead, due to its dense nature, inexpensive price, and superior shielding properties. Lead could seem like the perfect material for a shield, but its toxicity toward both people and the environment makes it less useful. The need for lead alternatives has increased recently, particularly in the medical industry, in an effort to curtail and eventually stop the use of lead as a shield. Glass is an additional substance that is employed as a radiation barrier. Glass has the special quality of being transparent as well as having a variety of compositions and being simple to manufacture [6,7,8,9,10,11].

Polymers represent a potentially attractive and acceptable option as shielding materials due to their exceptional chemical, physical, electrical, and radiation resistance capabilities, along with their flexibility, light weight, and durability. In addition, polymers can be efficiently doped with considerable quantities of high atomic number elements to form their composites, which are superior radiation shields [12]. Although polymers normally perform less well at radiation shielding than metals, their efficiency can be greatly increased by adding more radiation shielding chemicals to the polymer matrix [13]. Recently, polymer composites have drawn interest as viable, lightweight replacements for metal radiation protective technologies [14,15,16]. Polymers are multipurpose substances that are simple to mold for the intended applications. A single polymer cannot meet the criteria for technological usage in this area; hence, a number of factors must be taken into account. As a result, polymer composites have garnered interest on a global level.

Silicon rubber is often regarded as one of the polymers with a high degree of flexibility. This characteristic has a wide range of potential uses, particularly in the medical industry. As it can protect the body during a radiology evaluation, it must be enhanced with materials that have a higher density and can absorb photons. This will allow it to better serve its purpose [17,18]. Additives, such as heavy metal oxides and nanoparticles, may be incorporated into the polymers in order to enhance the rubbers’ capacity to shield electromagnetic radiation [19,20,21]. Typically, a bulk matrix and nano dimensions solid phase materials are combined to form nanocomposite materials. The nanocomposites made from polymers have benefits in terms of dimensional variation and flexibility. Radiation shielding is one application where polymers, composites, and polymeric nanocomposites are crucial. Materials made of polymer composites or nanocomposites have been used in a variety of industries, including in satellites, aerospace, nuclear reactors, etc. In any case, polymer composites have emerged as excellent prospects for the development of materials capable of attenuating photons or other types of particle radiation [22].

The macromolecular state around nanoparticles changes as a result of the high surface-to-volume ratio that distinguishes nanoparticles from other materials. The properties of the polymer are improved by the inclusion of nanoparticles, including improved elastic stiffness, strength, reduced gas permeability, and radiation shielding effectiveness [23]. Due to its excellent qualities and distinctive features, cerium oxide (CeO_2_) is attracting attention as a potential radiation shielding material. CeO_2_ is able to attenuate gamma photons because of its high atomic number. Radiation can be efficiently scattered and absorbed by the thick structure of CeO_2_, which reduces the amount of radiation that passes through the material. Moreover, CeO_2_ is ideal for long-term protection purposes due to its outstanding stability and radiation damage resistance. Furthermore, CeO_2_ is simply manufactured in a variety of shapes, such as bulk and nano-sized materials as well as thin films, providing versatility in the development and utilization of radiation shielding technologies [24,25]. Further research on CeO_2_’s potential as a protective material is required in the fields of radiation protection and nuclear security. For this reason, we prepared silicon rubber with micro and nano CeO_2_ and reported the radiation shielding properties of the prepared samples.

## 2. Materials and Method

**Figure 1:**
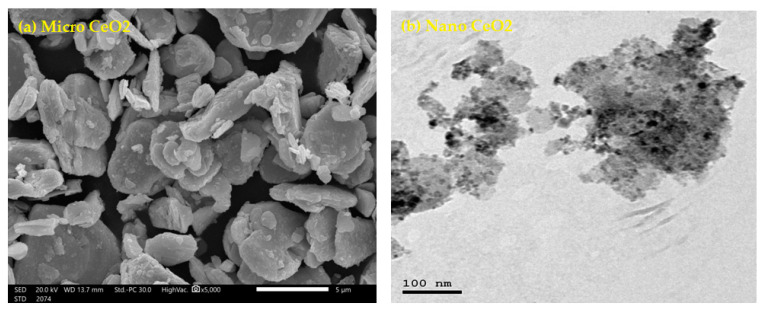
SEM and TEM images of micro and nano CeO_2_, respectively. (**a**) SEM of micro CeO_2_ and (**b**) TEM of nano CeO_2_.

We fabricated new flexible composites based on polydimethylsiloxane (this is a flexible polymer with interesting properties for biomedical applications, including physiological indifference, excellent resistance to biodegradation, biocompatibility, chemical stability, gas permeability, good mechanical properties, and excellent optical transparency) as a matrix material and cerium oxide (CeO_2_) with different particle sizes. The polydimethylsiloxane polymer was supplied from Alhuda Chemical in Egypt. The CeO_2_ was separated into 2 sizes, micro and nano. The average size of micro particles was 10 μm, supplied by Algamhoria Chemicals Company, while the CeO_2_ nanoparticle average size was 20 ± 3 nm, supplied from Nano Gate Company. The micro and nano CeO_2_ were supplied from Nano Gate Company. The average size of micro CeO_2_ was checked using an SEM scan as shown in Figure 1a, while the nano CeO_2_ was scanned by TEM analysis as shown in Figure 1b.

After collecting the basic materials, we prepared 10 different flexible SR samples, and each sample had different thicknesses (0.98, 1.35 and 2 cm). The weight percentage (%) ratio of SR to hardener was 95:5 in all preparations, and percentages of micro, nano, and 0.5 micro + 0.5 micro CeO_2_ are reported in Table 1. To obtain a homogeneous composite, the compounds were mixed with a hand mixer for a sufficient period equal to a quarter of an hour at room temperature, then placed in plastic crucibles of different thicknesses and left for 24 h until they became cohesive and flexible. The density of composites was measured and reported in Table 1 by evaluating the mass to volume ratio, where the mass was measured by 0.001 g sensitive electric balance and the volume measured by 43πr3, where r represents the radius of the SR composite [26].

**Figure 2:**
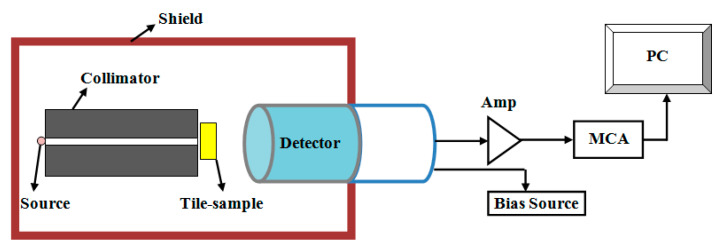
The experimental setup in the LAC measurement.

The attenuation or absorption parameters of these composites and the experimental LAC cm^−1^ were determined. The main devices used in the experimental technique were the point gamma ray sources, lead collimator, and HPGe detector connected to liquid nitrogen as well as an electronic unit containing a high voltage, multichannel analyzer connected to a computer, as shown in Figure 2. The detector was calibrated to get the best geometry for the measurements, and the free sample intensity of present sources was measured (I_0_). The sources were AM-241, Cs-137, and Co-60. Following this, the measurements were undertaken on an occupied SR sample to calculate the intensity (I) at a specific SR thickness (x). From these values, the experimental LAC can be calculated from the next relation [27,28,29]:

(1) $$LAC=\frac{1}{t}\ln\frac{I_{0}}{I}$$

To ensure the validity of the experimental values, the online Phy-X software was used to calculate the LAC of the pure SR composite. The Phy-X software can calculate the shielding values for photon energies between 0.015 MeV and 100 GeV for the molecular or elemental structure of any material [30]. The software accounts for the chemical makeup and density of the constituents while calculating these characteristics. Different shielding parameters, such as LAC, MFP, HVL, and TVL, can be calculated for each material. The relative deviation of 2 results is given by:

(2) $$RD\left(\%\right)=\frac{{LAC}_{Phy-x}-{LAC}_{Exp}}{{LAC}_{Exp}}*100$$

The relative increase between the composites containing micro and nano CeO_2_ (RI_1_) and the relative increase between the composites containing micro and (0.5 micro + 0.5 nano) CeO_2_ (RI_2_) were calculated as below:

(3) $${RI}_{1}\left(\%\right)=\frac{{LAC}_{Nano}-{LAC}_{Micro}}{{LAC}_{Micro}}\times 100$$

(4) $${RI}_{1}\left(\%\right)=\frac{{LAC}_{0.5N+0.5M}-{LAC}_{Micro}}{{LAC}_{Micro}}\times 100$$

The half value layer, 10th value layer, and radiation protection efficiency, denoted by HVL, TVL, and RPE, respectively, are essential factors for the shielding material properties and can be expressed by the following laws [31,32]:

(5) $$HVL=\frac{Ln(2)}{LAC}$$

(6) $$TVL=\frac{1}{LAC}$$

(7) $$RPE,\%=[1-\frac{I}{I_{0}}]\times 100$$

## 3. Results and Discussion

**Figure 3:**
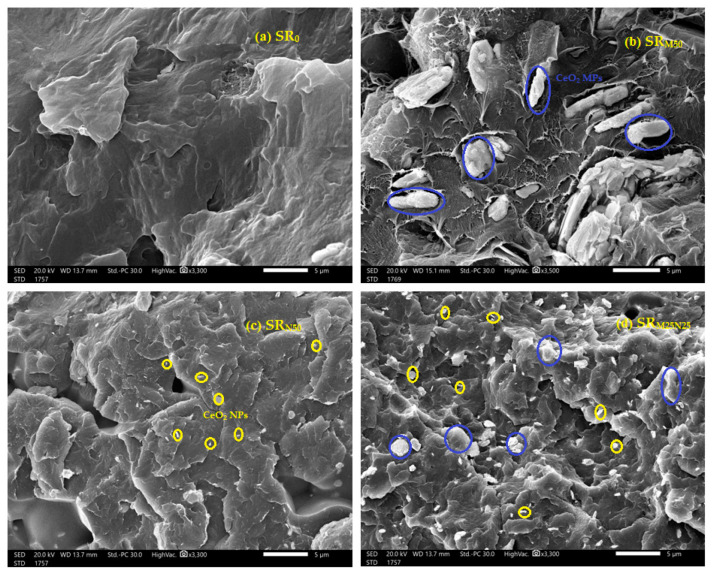
SEM images of the prepared fixable composites. (**a**) SR_0_, (**b**) SR_M50_, (**c**) SR_N50_, and (**d**) SR_M25N25_.

SEM morphologies of the SR_0_, SR_M50_, SR_N50_, and SR_M25N25_ composites were performed to observe the distribution of CeO2 size inside the silicon rubber, as shown in Figure 3. The results of the images show that the mixture of the micro and nano particles leads to a symmetrical and homogeneous distribution within the mixture, which reduces the voids between the silicon particles. This is evident in Figure 3d. The result of this good distribution makes the attenuation of the incident photons on the SR composite higher, which increases its shielding efficiency as shown below.

**Figure 4:**
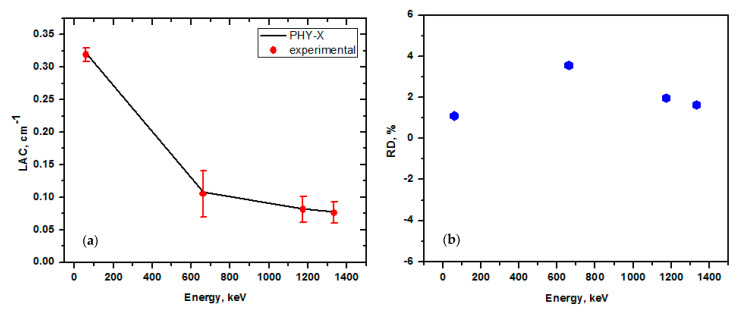
The experimental and theoretical LACs of the pure SR sample. (**a**) LAC and (**b**) relative deviation.

The purpose of this work was to analyze the radiation shielding capabilities of the synthesized SR and to assess the effects of various CeO_2_ contents, particle sizes (micro and nano) on the linear attenuation coefficient (LAC), and other related parameters. The first step is to test the accuracy in the setup used for the determination of the LAC for our samples. For this reason, we compared the experimental and Phy-X LAC for the pure SR samples. In Figure 4a, the LAC values determined by Phy-X are depicted in black squares, whereas the experimental LAC values are displayed in red circuits. Additionally, we display in Figure 4b the relative difference (R.D.) between the LACs for the pure SR sample obtained through experimental and theoretical methods. The R.D. represents the precision of the setup of the experiments employed in this study to calculate the LAC for the other kinds of samples (SR with micro and nano CeO_2_). The R.D. is minimal and falls within experimental uncertainty. Furthermore, as seen in Figure 3a, there is good agreement between the experimental and theoretical LACs, which shows that the experimental setup used in our work is appropriate for measuring the LAC of the SR with various CeO_2_ concentrations.

**Figure 5:**
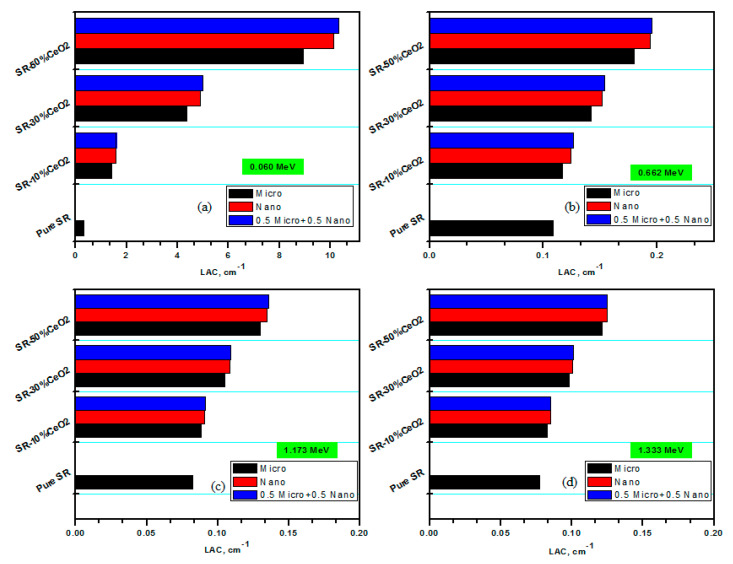
The LACs of different SR-CeO2 composites at different energies. (**a**) at 0.060 MeV, (**b**) at 0.662 MeV, (**c**) at 1.173 MeV, and (**d**) at 1.333 MeV.

In Figure 5, we presented the LACs for the pure SR and for the SR with 10, 30, and 50% of micro and nano CeO_2_. It is evident that regardless of whether the CeO_2_ is micro or nano, the LAC of the SR increases with increasing CeO_2_ concentration. This is due to the inclusion of the high atomic number elements (i.e., Ce with Z = 58).The pure SR has a lower LAC than all other SRs with CeO_2_. Therefore, increasing the amount of bulk CeO_2_ or NPs that are included within the SR can improve its ability to shield gamma photons. However, the speed at which the LAC increases is dependent on the energy of the photon that is being emitted. When CeO_2_ is added, the LAC value is shown to increase considerably at low energies; however, at high energies, the LAC value just slightly increases when CeO_2_ is added. This is due to the fact that the photoelectric effect is the most important process at low energy, and it is known that the cross section of this process highly depends on the atomic number of the materials. So, as we added CeO_2_, there was a notable increase in the LAC. However, at higher energies, the Compton scattering is the main photon–matter interaction process, and the cross section of this process has a weak dependence on the atomic number of the shielding materials, so we noticed a small increase in the LAC due to the addition of CeO_2_ at higher energies. The following section considers nano CeO_2_ and investigates how much the LAC increased for the lowest and highest measured energies.

The LAC for the pure SR at 0.059 MeV is 0.319 cm^−1^, while the LAC values are 1.56, 4.90, and 10.12 cm^−1^ for the SR with 10, 30, and 50% nano CeO_2_. As a consequence of this finding, the LAC improved as a direct result of the addition of 50% nano CeO_2_ to the SR. In the meantime, the values of the LAC for the pure SR, as well as the SR containing 10, 30, and 50% nano CeO_2_, were 0.076, 0.085, 0.101, and 0.125 cm^−1^, respectively, when measured at 1.333 MeV. As a result, the LAC at high energies exhibits only a slight increase as a result of the inclusion of nano CeO_2_, as can be easily observed in the results shown in Figure 5.

When the effect of the size of the particles on the LAC values is examined, it can be shown that the LACs for nano CeO_2_ are higher than that of micro CeO_2_, which appears as follows: (LAC) CeO_2_-nano > (LAC) CeO_2_-micro > (LAC)free CeO_2_. The particle distribution in the SR is the reason why the LAC values for CeO_2_ NPs are higher than those for micro CeO_2_. The NPs lower size enables a more uniform distribution of particles inside the SR, increasing the surface to mass ratio and raising the likelihood that gamma rays will interact with the CeO2 NPs. This is why the SR with NPs has superior attenuation capabilities than the SR with micro CeO_2_. Similar observation was revealed by Cheewasukhanonta et al., who examined the impact of nano and micro Bi_2_O_3_ on the attenuation factors for certain glass systems [33].

We prepared new samples that contain 5% micro CeO_2_ and 5 nano CeO_2_, 15% micro CeO_2_ and 15 nano CeO_2_, and 25% micro CeO_2_ and 25 nano CeO_2_. We used the abbreviation 0.5 M−0.5 N for these samples. We aimed to check the radiation shielding effectiveness of these samples that contain CeO_2_ in both sizes (i.e., micro and nano). Since the LACs for the nano CeO_2_ samples are higher than those of micro CeO_2_, we found that the LAC for the 0.5 M−0.5 N samples is higher than the LAC for the samples with nano CeO_2_, as shown in Figure 5. There are a variety of reasons why the LAC of the sample with both micro and nano CeO_2_ is higher than the LAC values for the samples with nano CeO_2_. First, the presence of both micro- and nano-sized CeO_2_ in the composite sample allows the SR to be more efficient at absorbing the incoming radiation. This enhancement can be attributed to the ability of different sized particles to interact with radiation from different energy ranges, leading to greater overall attenuation. Additionally, the distribution of particles within the composite sample may also cause this increase in LAC. Namely, the composite sample may have greater homogeneity compared to the sample with nano CeO_2_ because of the concentration of nanoparticles alongside the microparticles. This distribution of particles leads to more consistent attenuation of radiation across the samples.

**Figure 6:**
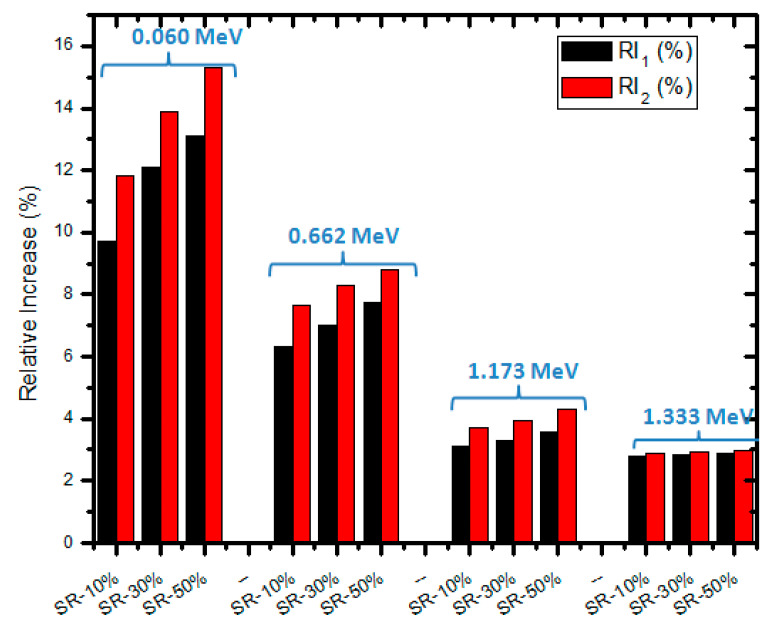
The relative increase between micro and nano as well as micro and 0.5 micro + 0.5 nano CeO_2_ at different energies.

The relative increase (RI, %) between the LAC values at each level of CeO_2_ was calculated in order to better understand the difference between the shielding effectiveness of micro and nano CeO_2_-SR (RI_1_) as well as the difference between the shielding effectiveness of micro and 0.5 micro + 0.5 nano CeO_2_-SR (RI_2_) against gamma photons. The ratios for the SR with 10, 30, and 50% CeO_2_ are displayed in a histogram in Figure 6. It is clear that as photon energy increases, the relative increase drops, highlighting how significantly CeO_2_ influences the SR ability to attenuate radiation at low energies. Additionally, it is evident that 30% CeO_2_ has a greater relative increase than 10% CeO_2_. The maximum RI (%) was reported for 50% CeO_2_ NPs at 0.059 MeV and is 31.10% and 15.30% for RI_1_ and RI_2_, respectively. Consequently, it can be stated that CeO_2_ nanoparticles are a more promising material to use in the development of efficient shielding materials than the micro particles, while the combination of both sizes of CeO_2_ gave better shielding development and a lower cost.

**Figure 7:**
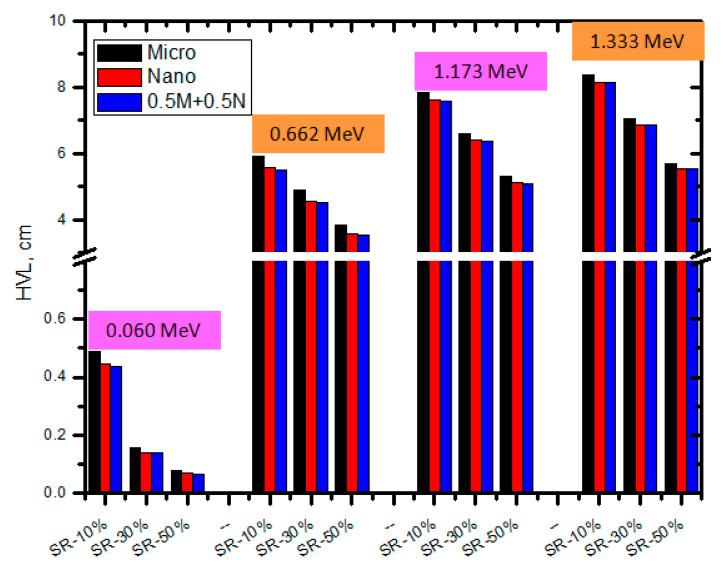
The HVL of SR with micro and nano CeO_2_ and the SR with CeO_2_ in both sizes at different energies.

The half value layers (HVL) of the SR with micro and nano CeO_2_ and the SR with CeO_2_ in both sizes (i.e., 0.5 M−0.5 N) at the examined energies were plotted in Figure 7. We observed that the HVL values of the SR with CeO_2_ were smaller than those of pure SR when comparing the HVL for the SR with micro and nano, indicating that the addition of CeO_2_ to the SR results in superior attenuation. The SR with 10% micro CeO_2_ has a greater HVL than the SR with 10% nano CeO_2_, as seen in Figure 6. In the meantime, SR with 30% micro CeO_2_ had a greater HVL than SR with 30% nano CeO_2_. This is due to the fact that the LAC for the SR with nano is higher than that of SR with micro CeO_2_ and it is known that the HVL has an inverse relation with the LAC according to the basic formula (HVL = 0.693/LAC). The same results are also correct for the SR with 50% CeO_2_. This finding demonstrated that the SR containing nanoparticles had a higher attenuation effectiveness than the SR with micro CeO_2_. When we compare the HVL of the SR with nano CeO_2_ and the HVL of SR with 0.5 M−0.5 N, we found that the HVL of the SR containing both sizes (micro and nano) is lower than the HVL of the SR with nano CeO_2_. For example, the HVL for SR with 10% nano CeO_2_ at 0.059 MeV is 0.443 cm, while it is 0.434 cm for the SR with 5% micro and 5% nano CeO_2_. For these two samples, the HVL at 0.662 MeV is 3.622 and 3.355 cm. This again confirms the importance of using both micro- and nano-sized CeO_2_ in the SR in order to improve the radiation shielding performance. Additionally, it is possible to note from Figure 7 that as the energy increases, the HVL increases for the pure SR in addition to the SR with micro CeO_2_ and nano CeO_2_; consequently, a thicker absorber is desirable in order to reduce the intensity of the radiation by half.

**Figure 8:**
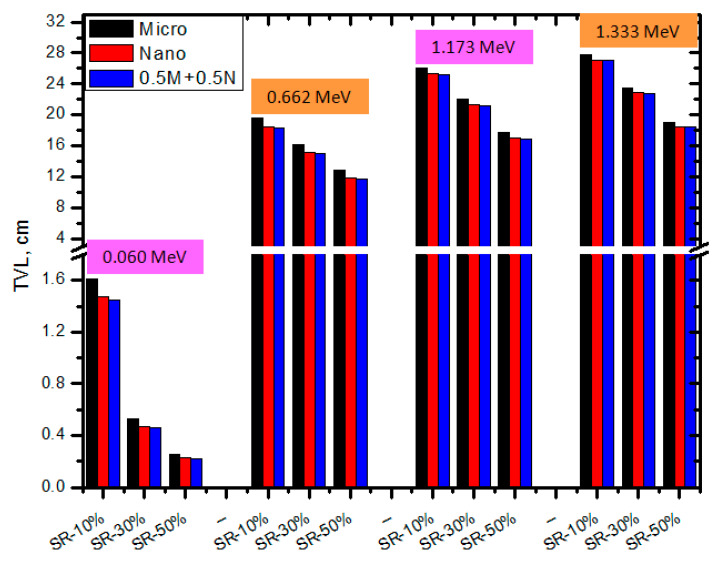
The TVL of SR with micro and nano CeO_2_ and the SR with CeO_2_ in both sizes at different energies.

Figure 8 displays, in a manner analogous to that of HVL, the 10th value layer (TVL) for the pure SR and the SR containing micro and nano CeO_2_, respectively. The TVL declined when CeO_2_ content was increased, as was expected, giving pure SR the highest TVL. Given that Ce is known to have a high atomic number, an increase in the likelihood of interactions between photons and the Ce atom could provide a reasonable interpretation for this tendency. The TVL values of the SR that contains nano CeO_2_ were lower than those containing micro CeO_2_ according to the analysis of the TVL for the SR doped with micro and nano CeO_2_. As a result, nano CeO_2_ is an effective photon shield. The HVL results and this outcome are consistent. These findings suggest that adding nano CeO_2_ to the SR significantly improves its shielding characteristics compared to using micro CeO_2_. When comparing the TVL for the SR with only nanoparticles and the SR with both micro and nanoparticles (i.e., 0.5 M−0.5 N), we found that the samples containing both sizes have a lower TVL than the SR with only nanoparticles. For example, the TVL for SR-10% nano CeO_2_ is 12.03 cm at 0.662 MeV, and it is 11.14 cm for the SR with 5% micro and 5% nano CeO_2_. It is clear from Figure 7 that the TVL is influenced by the energy of the radiation. Numerically, for the SR with 30% micro CeO_2_, the TVL increases from 1.61 to 19.65 cm between 0.059 and 0.662 MeV. We noticed the high difference in the TVL for the same sample between low energy (i.e., 0.059 MeV) and moderate energy (i.e., 0.662 MeV). For this composition, the maximum TVL is 27.85 cm, which was reported at 1.333 MeV.

**Figure 9:**
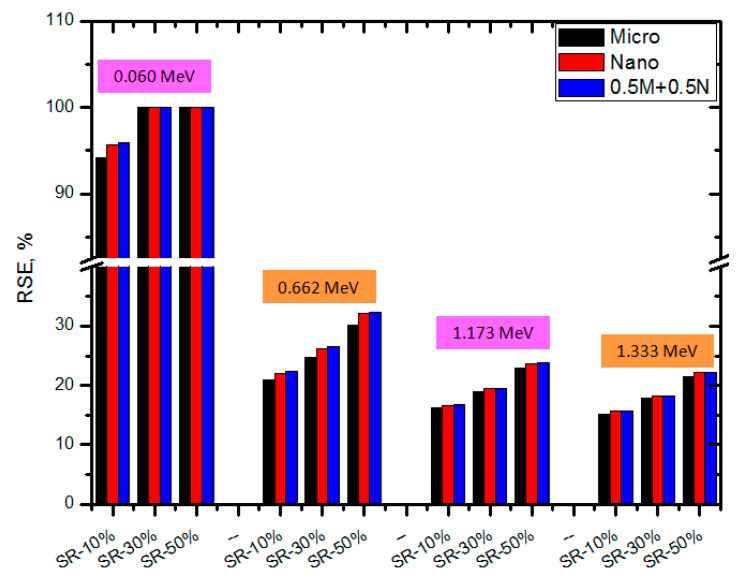
The RPE of SR with micro and nano CeO_2_ and the SR with CeO_2_ in both sizes at different energies.

Another advantageous parameter is the RPE. This gives explicit information about the efficacy of the particle size, which depends on the RPE of the manufactured SR. The findings of the RSE were presented in Figure 8 for the SR samples that had thicknesses of 2 cm. According to this statistic, pure SR has a lower RPE than any SR that contains micro or nano CeO_2_. This result indicates that integrating CeO_2_ into SR is a significant way to improve the shielding efficiency of the SR samples. This is due to the fact that Ce is a heavy element, and the addition of CeO_2_ to the SR will increase the likelihood that interactions will take place. In addition, looking at Figure 9 reveals that the RPE of the SR treated with nanoparticles is more than that of the SR treated with micro particles when both treatments were given at the same weight fraction. For example, at 0.059 MeV, the RPEs for the SR with 10% micro and nano CeO_2_ are 94.2 and 95.6%, respectively, while they are 20.9 and 31.8% for the same samples at 0.662 MeV. This result once again indicates the fact that the attenuation ability is improved when utilizing the nano CeO_2_ as opposed to the micro CeO_2_. In addition, the findings demonstrated that the SR samples exhibit their highest level of attenuation at 0.059 MeV. The RPE is almost 100% at 0.059 MeV, which means that the SR samples with micro or nano CeO_2_ can attenuate almost all the radiation with an energy of 0.059 MeV. The RPE at 0.662 MeV decreases 20–30% for the SR with micro CeO_2_, 31–47% for the SR with nano CeO_2_, and 34–49% for the samples with both micro and nano CeO_2_.

## 4. Conclusions

Research was conducted on the gamma-ray shielding capabilities of SR with micro and nano CeO_2_. The experimental approach was employed to find the LAC for each of the SR samples, and the results obtained from the SR containing micro CeO_2_ and the Phy-X software were completely consistent with each other. This suggests that the experimental nanoparticle findings were accurate. According to the findings, the LAC values for the nano CeO_2_ were greater than those for the micro CeO_2_ because the nano CeO_2_ had superior particle distribution in the SR. The nanoparticles’ lower size enables a more uniform distribution of the particles within the SR, increasing the surface to mass ratio and raising the likelihood of contact between the gamma rays and the nano CeO_2_. In addition, calculations were performed for shielding characteristics such as HVL and RPE. According to the findings, the HVL increases as the energy increases from 0.059 to 1.333 MeV for both pure SR and the SR samples with micro and nano CeO_2_. This suggests that an SR with a greater thickness is necessary in order to weaken photons with a high level of energy. The RPE results demonstrated that integrating CeO_2_ into SR is a significant way for improving the shielding efficiency of the produced SR samples. The RPE results revealed that the RPE of the SR treated with nanoparticles is greater than that of the SR treated with micro particles.

## Figures and Tables

**Table 1:** Codes, compositions, and densities of the different fabricated fixable samples.

Sample Name	Sample Code	SR (wt%)	CeO_2_ Micro (wt%)	CeO_2_ Nano (wt%)	Density (g·cm^−3^)
Pure SR	SR_0_	100	—	—	1.302 ± 0.006
SR with Micro CeO_2_	SR_M10_	90	10		1.410 ± 0.011
	SR_M30_	70	30		1.722 ± 0.010
	SR_M50_	50	50		2.201 ± 0.009
SR with Nano CeO_2_	SR_N10_	90		10	1.421 ± 0.010
	SR_N30_	70		30	1.742 ± 0.007
	SR_N50_	50		50	2.210 ± 0.008
SR with Micro and Nano CeO_2_	SR_M5N5_	90	5	5	1.416 ± 0.009
	SR_M15N15_	70	15	15	1.736 ± 0.007
	SR_M25N25_	50	25	25	2.213 ± 0.022

## Data Availability

All relevant data are within this paper.
